# Effects of recombinant *Echinococcus granulosus* antigen P29 on hematological indicators in sheep

**DOI:** 10.3389/fcimb.2026.1831004

**Published:** 2026-06-26

**Authors:** Hanyu Zhang, Yan Huo, Wei Huang, Jing Wang, Wei Zhao, Jihui Yang

**Affiliations:** 1 School of Basic Medicine, Ningxia Medical University, Yinchuan, China; 2 Ningxia Key Laboratory of Infection and Immunity, Ningxia Medical University, Yinchuan, China; 3 College of Resources, Environment and Life Sciences, Ningxia Normal University, Guyuan, China; 4 Department of Pathology, General Hospital of Ningxia Medical University, Yinchuan, China

**Keywords:** *Echinococcus granulosus*, immunization, recombinant antigen P29, sheep, vaccine

## Abstract

**Objective:**

This study aimed to evaluate the effects of immunization with recombinant *Echinococcus granulosus* antigen P29 (rEg.P29) on hematological, physiological, and biochemical indicators in sheep.

**Methods:**

A total of 36 4–6-month-old female Ningxia Yanchi Tan sheep, negative for *Brucella* and echinococcosis antibodies by ELISA, were randomly assigned to four groups (nine sheep per group). Following primary and booster immunizations with rEg.P29, peripheral blood samples were collected at weeks 0 and 20. ELISA, automated hematology analysis, and biochemical analysis were used to assess antigen-specific antibody levels, routine blood parameters, and biochemical indicators. At week 20 post-immunization, all sheep were humanely euthanized, and liver, spleen, and kidney tissues were collected for histopathological examination.

**Results:**

rEg.P29 significantly increased antigen-specific IgG levels at 20 weeks post-immunization compared with baseline. Hematological, biochemical, and histopathological analyses revealed no abnormal changes in blood parameters, physiological metabolism, or tissue morphology of the liver, spleen, and kidney, indicating a favorable safety profile.

**Conclusion:**

rEg.P29 immunization induces a robust humoral immune response without affecting hematopoietic cell homeostasis or liver and kidney function in sheep, as confirmed by hematological, biochemical, and histopathological analyses. These findings demonstrate the immunogenicity and safety of rEg.P29 and support its potential for the development of vaccines against echinococcosis.

## 1 Introduction

Echinococcosis, also known as hydatid disease, is a zoonotic parasitic disease that severely endangers human health and livestock husbandry, with a challenging global prevention and control situation (Rossi et al., 2016; Han et al., 2019; Anvari et al., 2021; Ma et al., 2021). Current therapeutic approaches mainly involve post-infection drug treatment or surgical intervention, both of which have inherent limitations (Al-Saeedi et al., 2021; Wan et al., 2021). Vaccine-based prevention has emerged as a promising control strategy (Moghaddam et al., 2019; Zhao et al., 2023; Jiang et al., 2025). The recombinant vaccine developed by our research team exhibited excellent immunoprotective potential (Zhang et al., 2024), with protection rates of 96.6% and 94.8% in mouse (Shi et al., 2009) and sheep (Wang et al., 2016) models, respectively, demonstrating strong potential for further vaccine development.

In vaccine evaluation systems, changes in hematological indicators provide a critical window for comprehensively assessing vaccine efficacy (Li et al., 2022; Aldali et al., 2023). Immune responses induced by vaccination are directly or indirectly reflected in blood parameters, which indicate immune cell activation and overall physiological status, whereas blood biochemical indicators reflect protein metabolism, liver function, and kidney function. These hematological parameters not only help evaluate vaccine immunogenicity but also support safety monitoring. Sheep are the primary intermediate hosts of echinococcosis (Borhani et al., 2024; Poggio et al., 2024) and exhibit a much higher infection rate than other livestock species. During disease transmission, sheep become infected by ingesting food or water contaminated with feces from definitive hosts and may further contribute to human infection through environmental contamination. As a common livestock species widely raised in Northwest China (Gao et al., 2022), sheep play a pivotal role in the transmission of echinococcosis, making quarantine (Rong et al., 2021; Qi et al., 2024) and monitoring essential for disease control. However, systematic research on the effects of the recombinant *Echinococcus granulosus* antigen P29 (rEg.P29) vaccine on hematological indicators in sheep remains limited.

Therefore, this study monitored changes in peripheral blood cell counts, differentiation, and serum biochemical indicators in sheep following rEg.P29 immunization, aiming to evaluate the vaccine’s immunological efficacy and safety from a hematological perspective and to provide comprehensive experimental data for the subsequent development and application of vaccines against echinococcosis.

## 2 Materials and methods

### 2.1 Preparation of rEg.P29

The antigen used in this study was a recombinant P29 gene-containing plasmid constructed and preserved in our laboratory, and the rEg.P29 protein was expressed using the pET28a (+) vector in *Escherichia coli* BL21(DE3) cells. The complete amino acid sequence of rEg.P29 was obtained from GenBank (accession number XP_024351425.1), and the recombinant antigen P29 consists of 238 amino acids. Expression and purification of the rEg.P29 protein were performed according to previously established protocols. Briefly, identified bacterial strains were inoculated into Luria–Bertani (LB) liquid medium containing 0.1 mM isopropyl-β-D-thiogalactopyranoside (IPTG; Invitrogen, USA) and induced at 37 °C for 10 h. After bacterial collection, recombinant proteins were purified using a Histidine-Tagged Protein Purification Kit (Merck, USA) according to the manufacturer’s instructions. The recombinant antigen P29 is a soluble protein and was purified by NiNTA affinity chromatography using binding buffer (20 mM Tris-HCl, pH 8.0, 500 mM NaCl, 5 mM imidazole), washing buffer (20 mM Tris-HCl, pH 8.0, 500 mM NaCl, 20 mM imidazole), and elution buffer (20 mM Tris-HCl, pH 8.0, 500 mM NaCl, 250 mM imidazole). To eliminate endotoxin interference in subsequent animal experiments, the purified protein solution was treated using an Endotoxin Removal Kit (GenScript, Nanjing, China). Finally, the purity and molecular weight of the purified protein were verified by SDS-PAGE, and the purity exceeded 98%. The antigen concentration of rEg.P29 was determined using a BCA Protein Assay Kit (KeyGen Biotech, Nanjing, China).

### 2.2 Animal immunization

**Figure 1:**
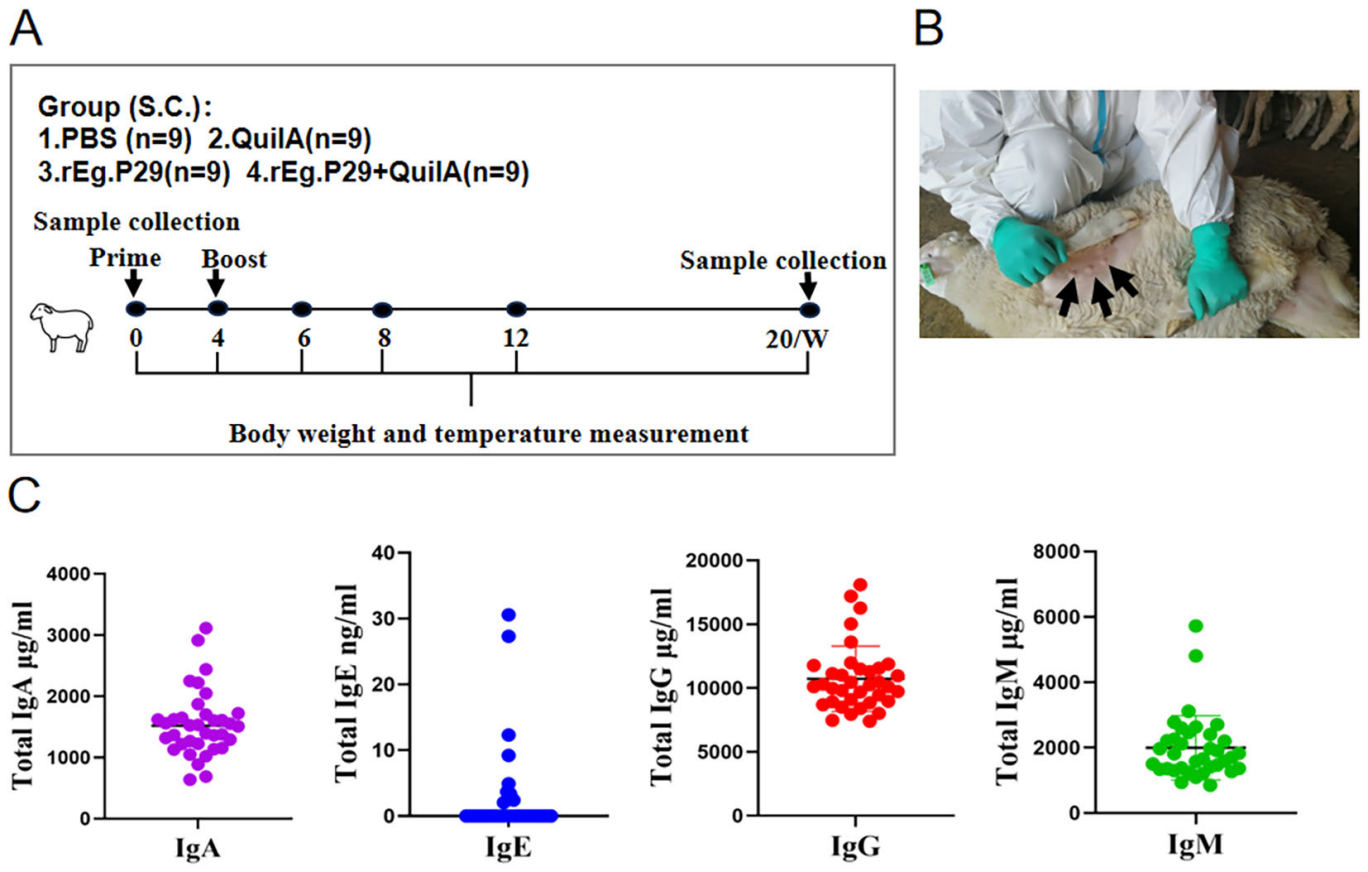
Immunization strategy and determination of serum antibody levels in sheep **(A)** Scheme of subcutaneous immunization and sample collection in sheep (n = 9 per group). Sheep were immunized via subcutaneous injection in the abdomen, with a 4-week interval between the primary and booster immunizations. Peripheral blood samples were collected at weeks 0 and 20 for serum antibody detection. Peripheral blood samples collected at week 20 were also used for blood routine and biochemical tests. Body weight and body temperature were measured at weeks 0, 4, 6, 8, 12, and 20 after immunization. Groups included the PBS group, QuilA group, rEg.P29 group, and rEg.P29 + QuilA group (n = 9 per group). **(B)** Sheep were immunized at three different subcutaneous sites (indicated by black arrows). **(C)** Serum levels of total serum IgA, IgE, IgG, and IgM in sheep before immunization. Results are presented as mean ± SD.

A total of 36 4–6-month-old female Ningxia Yanchi Tan sheep that tested negative for *Brucella* and echinococcosis antibodies by ELISA (to avoid infection risks and interference with immunization results; **Table 1**) were randomly divided into four groups (nine sheep per group). Primary and booster immunizations were administered via subcutaneous injections at three sites (**Figure 1B**) at weeks 0 and 4, respectively (**Figure 1A**). The PBS control group received 1 mL sterile PBS; the Quil A adjuvant control group received 1 mg Quil A adjuvant solution; the rEg.P29 immunization group received 50 μg rEg.P29; and the rEg.P29 + Quil A immunization group received 50 μg rEg.P29 plus 1 mg Quil A adjuvant.

**Table 1:** Basic information of sheep used in this study, including sex, age, breed, body weight, and detection results for *Brucella* and *Echinococcus granulosus* pathogens.

No.	Ewe/ram	Age (months)	Species	Weight (kg)	*Brucella*	*Echinococcosis*
1	Ewe	4–6	Chinese Yan chi Tan	25.2	-	-
2	Ewe	4–6	Chinese Yan chi Tan	22.4	-	-
3	Ewe	4–6	Chinese Yan chi Tan	20.4	-	-
4	Ewe	4–6	Chinese Yan chi Tan	27.5	-	-
5	Ewe	4–6	Chinese Yan chi Tan	22.7	-	-
6	Ewe	4–6	Chinese Yan chi Tan	22.8	-	-
7	Ewe	4–6	Chinese Yan chi Tan	23.8	-	-
8	Ewe	4–6	Chinese Yan chi Tan	20.4	-	-
9	Ewe	4–6	Chinese Yan chi Tan	23.0	-	-
10	Ewe	4–6	Chinese Yan chi Tan	21.1	-	-
11	Ewe	4–6	Chinese Yan chi Tan	21.0	-	-
12	Ewe	4–6	Chinese Yan chi Tan	23.8	-	-
13	Ewe	4–6	Chinese Yan chi Tan	21.9	-	-
14	Ewe	4–6	Chinese Yan chi Tan	25.7	-	-
15	Ewe	4–6	Chinese Yan chi Tan	26.0	-	-
16	Ewe	4–6	Chinese Yan chi Tan	22.4	-	-
17	Ewe	4–6	Chinese Yan chi Tan	25.7	-	-
18	Ewe	4–6	Chinese Yan chi Tan	25.8	-	-
19	Ewe	4–6	Chinese Yan chi Tan	23.8	-	-
20	Ewe	4–6	Chinese Yan chi Tan	22.2	-	-
21	Ewe	4–6	Chinese Yan chi Tan	23.2	-	-
22	Ewe	4–6	Chinese Yan chi Tan	19.8	-	-
23	Ewe	4–6	Chinese Yan chi Tan	19.8	-	-
24	Ewe	4–6	Chinese Yan chi Tan	22.3	-	-
25	Ewe	4–6	Chinese Yan chi Tan	27.5	-	-
26	Ewe	4–6	Chinese Yan chi Tan	22.7	-	-
27	Ewe	4–6	Chinese Yan chi Tan	24.3	-	-
28	Ewe	4–6	Chinese Yan chi Tan	24.0	-	-
29	Ewe	4–6	Chinese Yan chi Tan	23.5	-	-
30	Ewe	4–6	Chinese Yan chi Tan	28.5	-	-
31	Ewe	4–6	Chinese Yan chi Tan	20.9	-	-
32	Ewe	4–6	Chinese Yan chi Tan	23.0	-	-
33	Ewe	4–6	Chinese Yan chi Tan	24.0	-	-
34	Ewe	4–6	Chinese Yan chi Tan	24.7	-	-
35	Ewe	4–6	Chinese Yan chi Tan	21.2	-	-
36	Ewe	4–6	Chinese Yan chi Tan	24.0	-	-

### 2.3 Sample collection and pretreatment

Peripheral blood samples were collected from the jugular vein at week 20. Blood samples were immediately transferred to vacuum tubes containing EDTA-K2 anticoagulant, gently inverted 5–8 times, and analyzed within 2 h. Simultaneously, blood was collected into vacuum coagulation-promoting tubes without anticoagulant, incubated at 37 °C for 30 min to allow complete coagulation, and centrifuged at 1,000 × g for 10 min to separate serum. The serum was aliquoted and stored at -80 °C for biochemical analysis. Peripheral blood was also collected from the jugular vein at weeks 0 and 20, and serum was isolated for the detection of specific antibodies.

### 2.4 Measurement of animal body weight and body temperature

Body weight and body temperature were recorded at weeks 0, 4, 6, 8, 12, and 20. Rectal temperature measurements were performed as follows: the mercury column of the thermometer was shaken down to below 35 °C, disinfected, lubricated, and gently inserted into the rectum to approximately two-thirds of its length. The thermometer was retained for 3–5 min, then removed, cleaned and read.

### 2.5 Enzyme-linked immunosorbent assay

ELISA was used to detect total serum antibodies before immunization and anti-rEg.P29-specific antibodies after immunization. Quantitative detection of IgA, IgE, IgG, and IgM in pre-immunization serum was performed using ELISA kits (Huamei Biotechnology Co., Ltd., Wuhan, China) according to the manufacturer’s protocols. Standard curves were generated according to the kit standards, and antibody concentrations were calculated accordingly. Peripheral blood samples were incubated at 37 °C for 1 h and centrifuged at 12,000 × g for 5 min to obtain serum. Reagents, standards, and enzyme-labeled secondary antibodies were equilibrated at room temperature for 1 h. Washing buffer (25×) was diluted 25-fold with distilled water to prepare the working solution. Samples were added to 96-well microplates, sealed, and incubated at 37 °C for 1 h. The plates were washed five times with PBST and dried. Then, 100 μL of HRP-labeled secondary antibody was added to each well, and the plates were sealed and incubated at 37 °C for 30 min. After washing, 100 μL of TMB substrate solution (3,3’,5,5’-tetramethylbenzidine) was added and incubated in the dark at room temperature for 15 min. The reaction was terminated with 50 μL of 2 M H_2_SO_4_, and absorbance was measured at 450 nm using a Multiskan SkyHigh microplate spectrophotometer.

For post-immunization detection of specific antibodies, rEg.P29 was diluted to 5 μg/mL in carbonate buffer (pH 9.6), coated onto 96-well microplates, and incubated overnight at 4 °C. Plates were washed five times with PBST (PBS containing 0.05% Tween-20) and blocked with 5% non-fat milk at 37 °C for 2 h. After washing, diluted sheep serum was added as the primary antibody and incubated at 37 °C for 1 h. HRP-conjugated anti-sheep immunoglobulins (IgG, IgM, IgA, and IgE; ABD Serotec, Kidlington, UK) were added and incubated. The plates were washed again and incubated at 37 °C for 1 h, after which the TMB substrate solution was added for the chromogenic reaction. The reaction was terminated with 2 M H_2_SO_4_, and absorbance was measured at 450 nm using a Multiskan SkyHigh microplate spectrophotometer (Thermo Fisher Scientific, Massachusetts, USA).

### 2.6 Blood routine analysis

Whole blood samples collected in EDTA-K2 anticoagulant tubes were analyzed within 2 h using an automatic hematology analyzer (XS-900i, Sysmex Corporation, Kobe, Japan). After automatic mixing, the following parameters were measured: red blood cell count, hemoglobin concentration, hematocrit, platelet count, white blood cell count, and a five-part differential leukocyte count. Each sample was analyzed in duplicate, and the average value was recorded.

### 2.7 Biochemical indicator detection

Serum samples obtained from coagulation-promoting tubes were analyzed using an automatic biochemical analyzer (DXC700AU, Beckman Coulter, Brea, CA, USA) according to the manufacturer’s instructions. Key indicators included total protein, albumin, globulin, liver function markers (ALT and AST), and kidney function markers (CREA and BUN).

### 2.8 Histopathological examination

At 20 weeks post-immunization, sheep were sedated with an intravenous injection of propofol (20 mg/kg) and subsequently euthanized by intravenous administration of potassium chloride (100 mg/kg). Tissue samples from the liver, spleen, and kidney were collected promptly, fixed in 4% paraformaldehyde for 24–48 h, and then subjected to routine dehydration and paraffin embedding. Serial sections were cut at a thickness of 5 μm and stained with hematoxylin and eosin (H&E) using a standard protocol (hematoxylin for 5 min and eosin counterstaining for 3 min). Histopathological changes were observed under a light microscope at 20× magnification.

### 2.9 Statistical analysis

Experimental data were recorded in Excel and analyzed using SPSS version 22.0 (IBM, USA) and GraphPad Prism 8.0 (GraphPad Software Inc., USA). All data are presented as the mean ± standard deviation (SD). The Shapiro–Wilk test was used to assess data normality, and Levene’s test was performed to evaluate the homogeneity of variances. Two-way analysis of variance (ANOVA) was performed to evaluate the effects of treatment and time, followed by Tukey’s multiple comparison test for *post-hoc* analysis. Exact P values are reported. A P value of less than 0.05 was considered statistically significant, while a P value over 0.05 indicated no significant difference between groups.

## 3 Results

### 3.1 Consistency of serum antibody levels in sheep

To clarify the baseline immune status of the sheep, pre-immunization serum antibodies (IgA, IgM, IgE, and IgG) were measured by ELISA, and standard curves were generated to calculate antibody concentrations. As shown in **Figure 1C**, IgG had the highest total concentration (10723.4 ± 2524.09 μg/mL), whereas IgE had the lowest (15.4 ± 31.20 ng/mL). A high baseline IgG level reflects the physiological IgG pool in sheep, including maternal antibodies and natural antibodies against environmental antigens. IgA and IgM levels were intermediate, with IgM (1992.4 ± 971.69 μg/mL) slightly higher than IgA (1544.3 ± 528.81 μg/mL). These results indicate that antibody levels were relatively uniform, confirming a consistent baseline immune status among the 36 sheep.

### 3.2 rEg.P29 does not affect sheep body weight or body temperature

**Figure 2:**
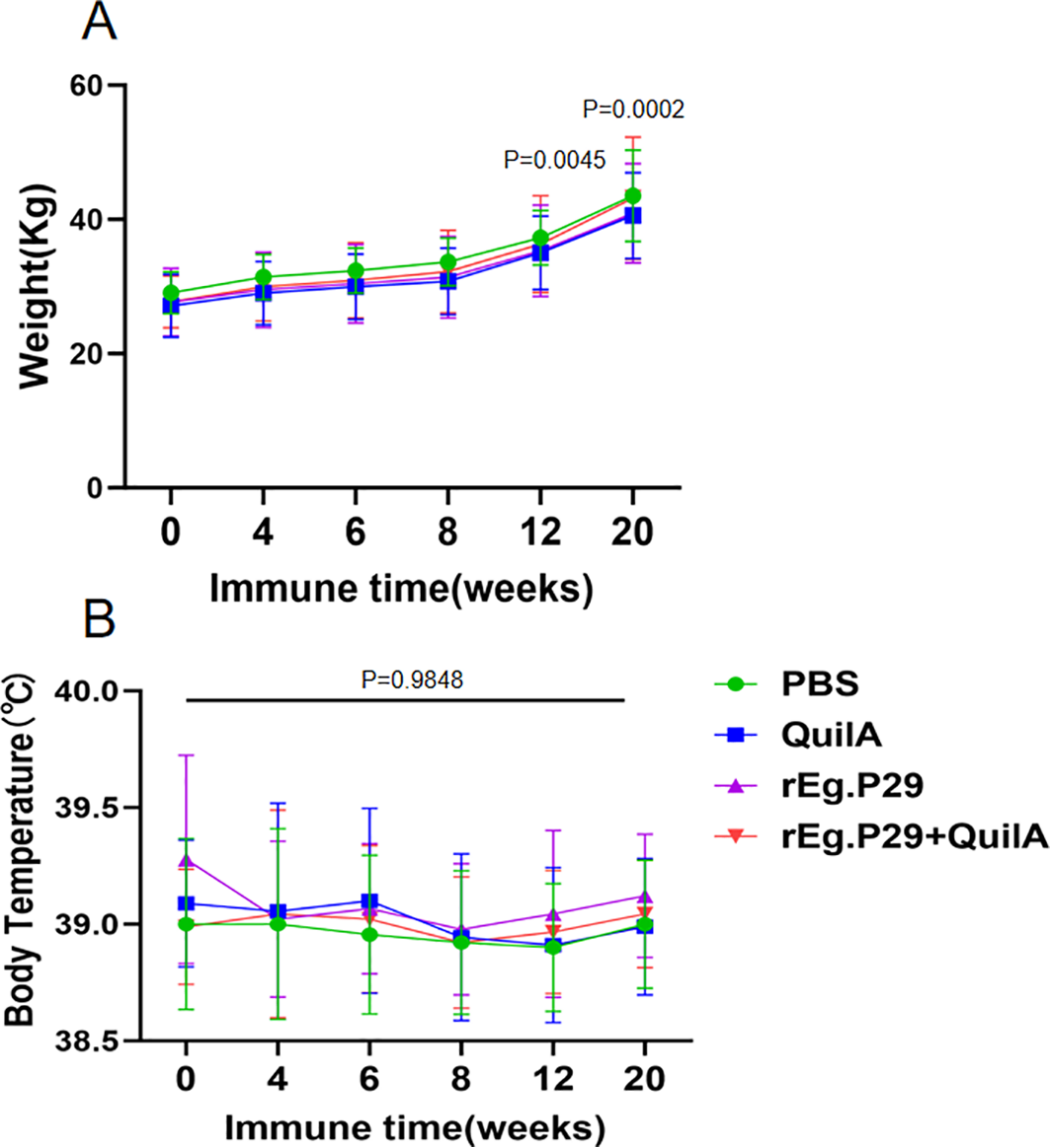
Changes in body weight and body temperature of sheep. **(A)** Body weight of sheep in each group at different time points. **(B)** Body temperature of sheep in each group at different time points. Significant differences in body weight were observed at week 12 (P = 0.0045) and week 20 (P = 0.0002); no significant difference was found in body temperature (P = 0.9848). Results are presented as mean ± SD.

Body weight and body temperature serve as quantitative indicators of vaccine immunogenicity and safety. Changes in body weight reflect the appropriateness of the antigen/adjuvant dosage (ensuring comparability between groups), whereas changes in body temperature indicate inflammatory responses and immune reaction intensity. Dynamic monitoring of the four groups (**Figure 2**) showed that body weight increased consistently across all groups, with no abnormal elevation or reduction, indicating normal growth and no significant inhibitory effects of the vaccine. Although differences in weight were observed at later time points (e.g., weeks 12 and 20), the overall growth trend remained consistent. Body temperature remained within the normal range for sheep (38.5 °C–39.5 °C), with no significant differences between groups, confirming the absence of overt acute inflammatory responses or immune stress induced by immunization.

### 3.3 rEg.P29 induces sustained humoral immune responses in sheep

**Figure 3:**
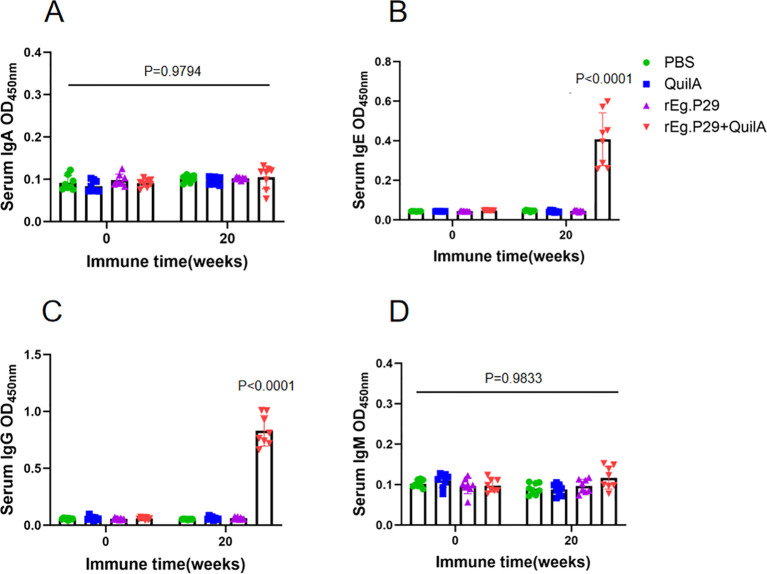
Expression of antigen-specific antibodies in serum. **(A)** Serum IgA expression. **(B)** Serum IgE expression. **(C)** Serum IgG expression. **(D)** Serum IgM expression. Peripheral blood samples were collected at week 0 (pre-immunization) and week 20 post-immunization, and serum was isolated for antibody detection. Experimental groups included the PBS control group, QuilA adjuvant group, rEg.P29 group, and rEg.P29 + QuilA group (n = 9 per group). Statistical analysis was performed using two-way ANOVA followed by Tukey’s multiple comparison test. Exact P values for pairwise comparisons are indicated in the figure. At week 20 post-immunization, serum IgE and IgG levels in the rEg.P29 + QuilA group were significantly higher than those in the other three groups (P < 0.0001). No significant differences were observed in serum IgA and IgM levels among the groups (P > 0.05). Results are presented as mean ± SD.

To evaluate the humoral immune responses, serum-specific antibody levels were monitored following rEg.P29 immunization. As shown in **Figure 3**, rEg.P29 alone did not induce a significant increase in specific IgG at 20 weeks post-immunization, whereas the formulation with Quil A adjuvant elicited a highly significant increase in rEg.P29-specific IgG (*P* < 0.0001), confirming that Quil A is essential for inducing a robust IgG-dominant humoral immune response in sheep. A significant increase in specific IgE (*P* < 0.0001) was also observed in the adjuvant-supplemented group, whereas no significant changes were detected in specific IgM or IgA (ns, *P* > 0.05).

### 3.4 rEg.P29 does not alter blood routine indicators

**Figure 4:**
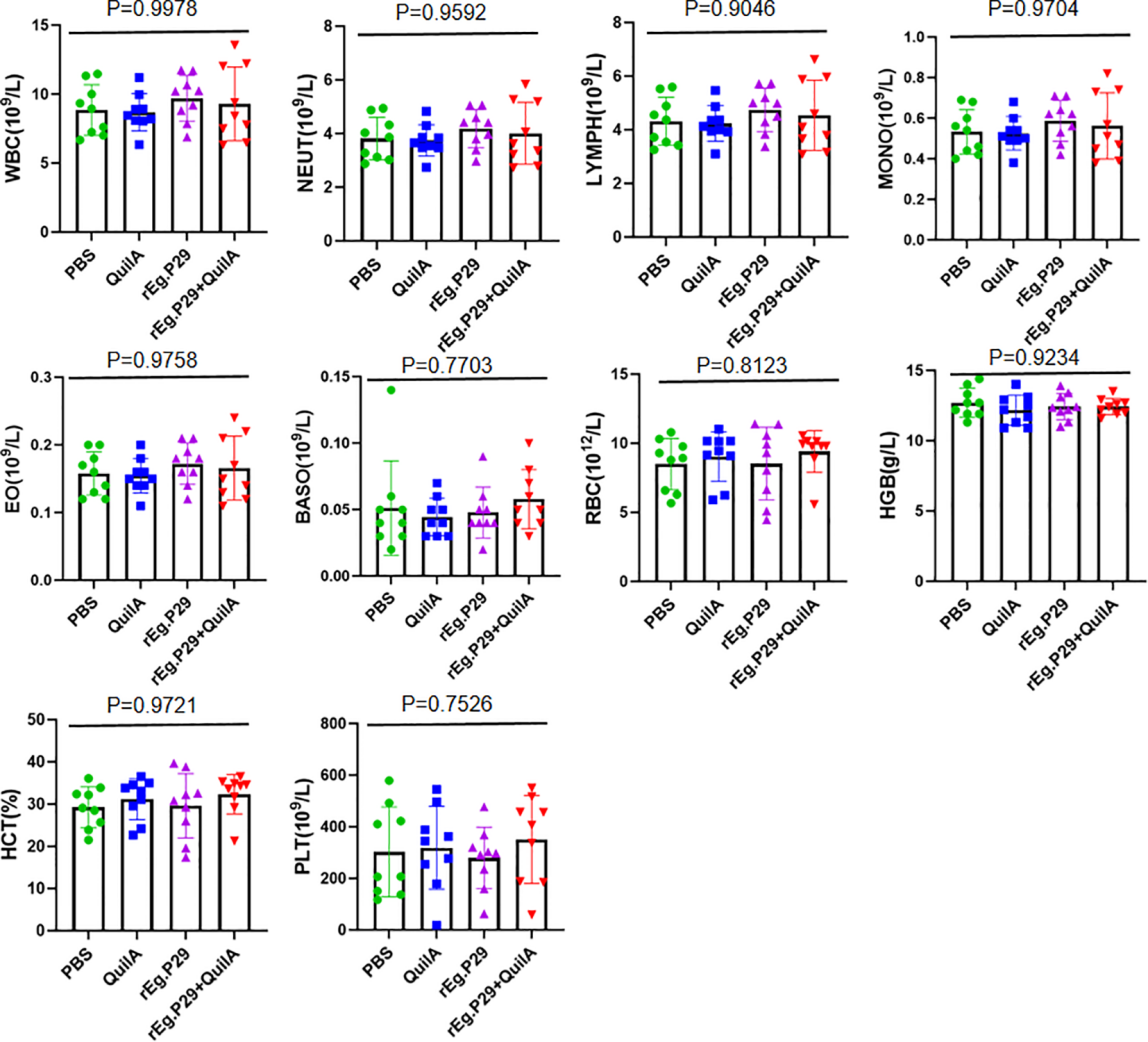
Blood routine results of sheep peripheral blood. Peripheral blood samples were collected from all groups at week 20, and blood routine parameters were measured using a hematology analyzer. Exact P values for pairwise comparisons are indicated in the figure. No significant differences were observed in any hematological parameter among the four groups (all P>0.05). Results are presented as mean ± SD.

Routine blood parameters, including red blood cells, white blood cells, and platelets, reflect physiological status, inflammatory responses, and immune activation. At week 20 following primary and booster immunizations, routine blood analysis (**Figure 4**) showed no statistically significant differences between the rEg.P29 + Quil A group and the control groups (PBS and Quil A) in immune-related cell subsets (white blood cell count and neutrophil/lymphocyte percentages) or in red blood cell and platelet parameters. All values remained within normal physiological ranges. These findings indicate that rEg.P29 immunization stimulates specific immune responses without abnormal fluctuations, suggesting a precise immune process without non-specific inflammatory signals (e.g., bacterial infection or tissue damage) and no adverse effects on baseline hematological physiology, thereby supporting its safety.

### 3.5 rEg.P29 does not affect biochemical indicators

**Figure 5:**
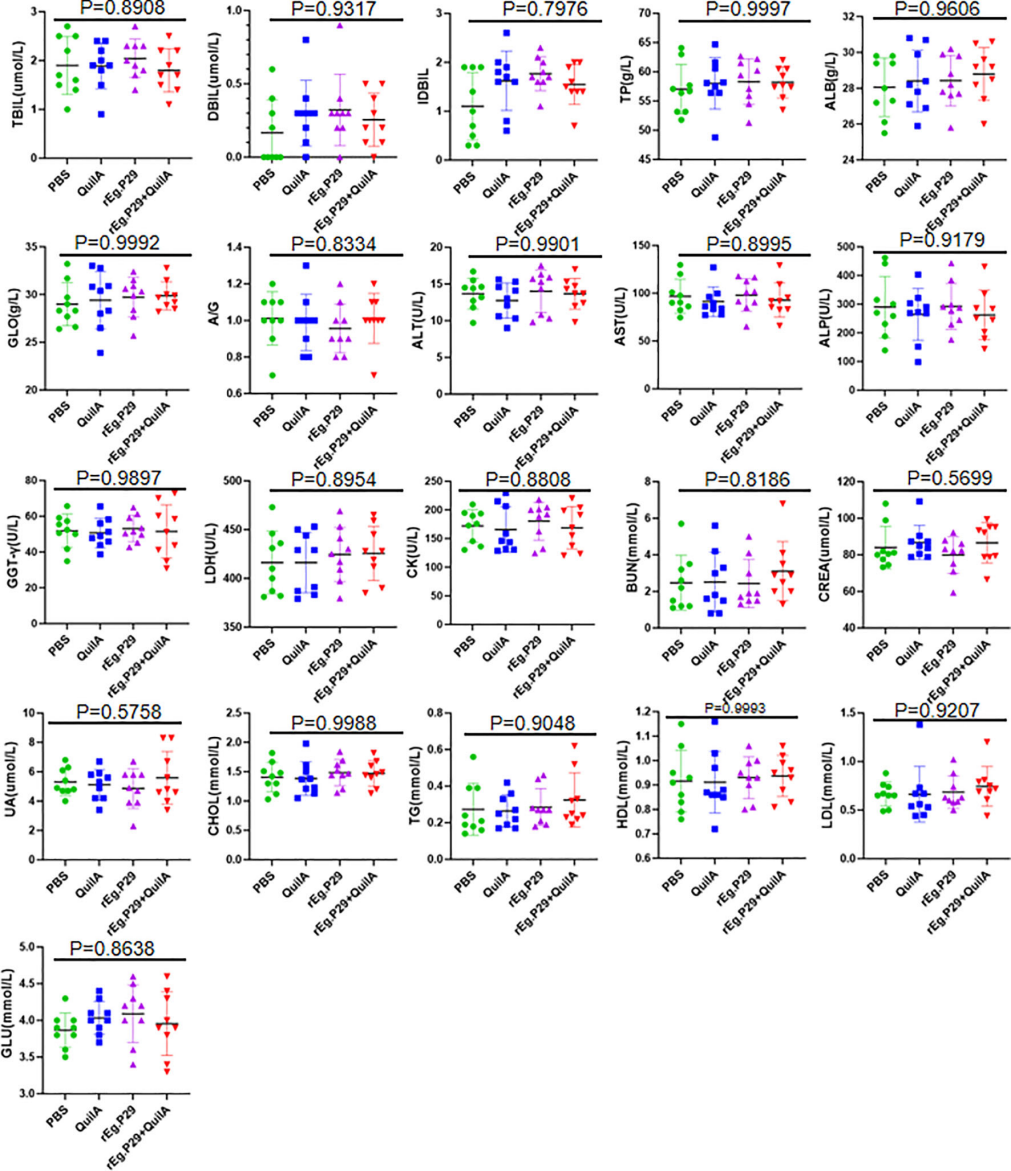
Biochemical index results of sheep peripheral blood. Peripheral blood samples were collected from all groups at week 20; serum was separated, and the serum biochemical indices were detected using an automatic biochemical analyzer. Exact P values for pairwise comparisons are indicated in the figure. No significant differences were observed in any serum biochemical parameters among the four groups (all P > 0.05). Results are presented as mean ± SD.

Biochemical indicators (liver function, kidney function, and protein metabolism) were analyzed to assess vaccine-induced systemic effects. At week 20 post-immunization, serum biochemical analysis (**Figure 5**) showed a non-significant trend toward increased total protein and globulin levels in the rEg.P29 + Quil A group, consistent with elevated specific antibody levels. This trend may be attributable to limited sample size, individual variation, or analytical variability. Liver function indicators (ALT and AST) remained within normal ranges, with no significant differences between groups, indicating no detectable liver damage during the experiment. Kidney function parameters (CREA and BUN) also showed no significant changes and remained within normal physiological limits, excluding evidence of immune complex-mediated renal impairment. Collectively, these results indicate that rEg.P29 immunization elicits immune responses without observable liver or kidney damage, further supporting its safety.

### 3.6 rEg.P29 did not induce pathological damage to sheep organs

**Figure 6:**
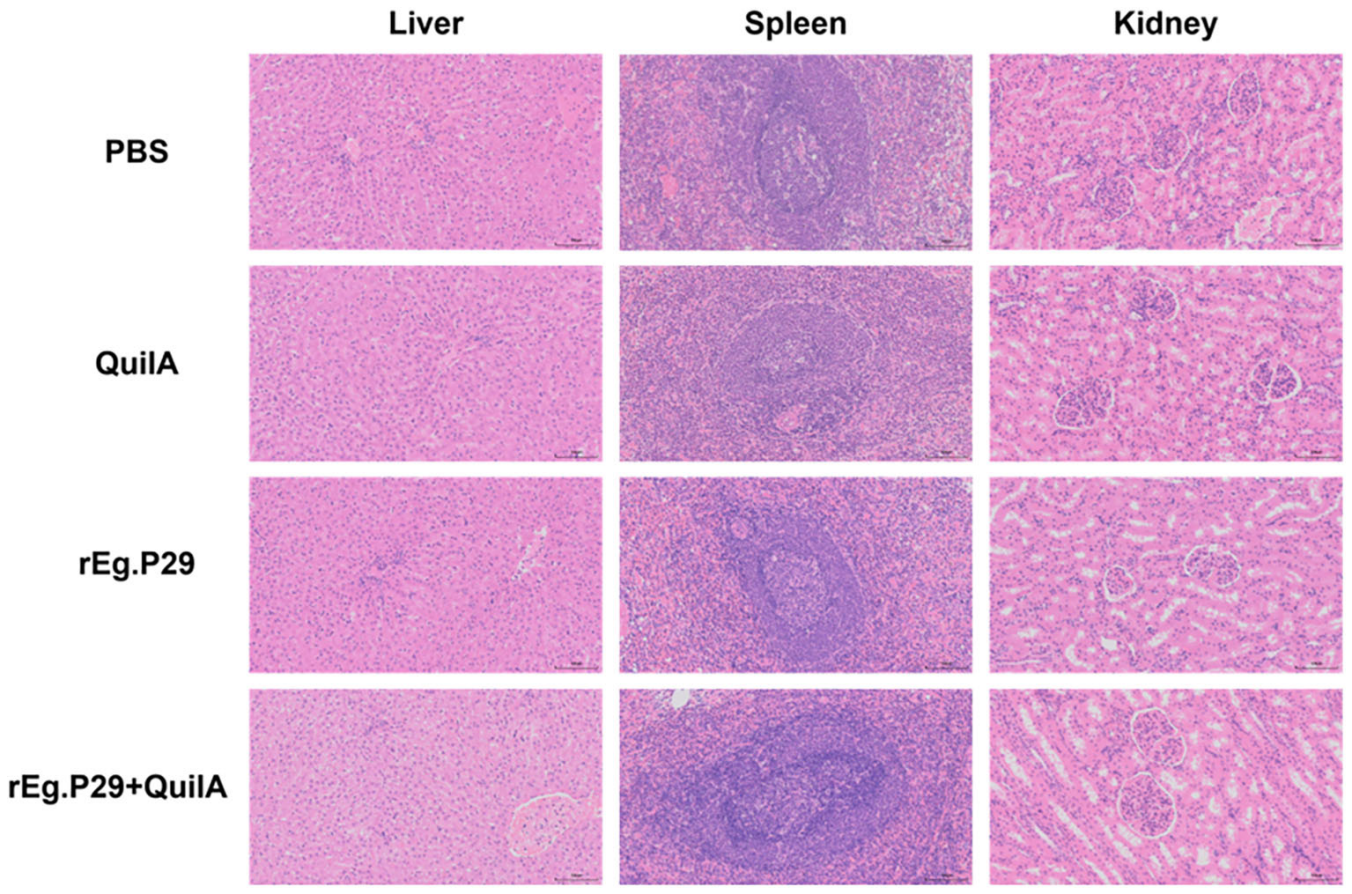
Histopathological observation of liver, spleen, and kidney tissues from immunized sheep (H&E staining, ×20 magnification, scale bar = 100 μm). Sheep were immunized with PBS, Quil A, rEg.P29, or rEg.P29 + Quil A. Tissue sections were stained with hematoxylin and eosin (H&E) and examined under a light microscope at ×20 magnification (scale bar = 100 μm).

In this study, morphological changes in the liver, spleen, and kidney tissues of immunized sheep were assessed by H&E staining. As shown in **Figure 6**, compared with the PBS control group, the QuilA group, rEg.P29 group, and rEg.P29 + QuilA group all exhibited normal tissue morphology in the liver, spleen, and kidney. The hepatic lobules maintained an intact structure with regularly arranged hepatocytes. In the spleen, the boundary between the white pulp and red pulp was clear, and the tissue structure remained intact. Notably, in the rEg.P29 + QuilA co-immunization group, the splenic white pulp showed extensive hyperplasia, with densely packed lymphoid follicles that were markedly enlarged. No degeneration was observed in the glomeruli or renal tubules, and the renal tissue structure was normal. In the rEg.P29 + QuilA group, no pathological changes, such as inflammatory cell infiltration, tissue necrosis, or structural disruption, were detected in any of the examined organs, with no significant differences observed compared to the control group.

## 4 Discussion

Sheep are the primary intermediate hosts of echinococcosis (Wen et al., 2019) and serve as a fundamental means of subsistence and an important asset for people in pastoral areas. *Echinococcus* larvae can parasitize the liver, lungs, and other organs of sheep, which not only inhibit growth and may lead to death but can also be transmitted to humans through the fecal–oral route, contributing to the spread of parasitic diseases (Gao et al., 2021; Hua et al., 2022; Huo et al., 2025) and posing a significant threat to public health (Yang et al., 2025). In animal husbandry and pastoral communities, sheep health is directly related to household income, economic stability, and livelihood security. Therefore, the development of an echinococcosis vaccine with favorable immunogenicity, safety, and tolerability is imperative. In this study, 36 female Ningxia Yanchi Tan sheep aged 4–6 months with similar body weights and negative Brucella/echinococcosis antibody status were immunized with rEg.P29 (primary and booster immunization). Systematic evaluation of physiological parameters and antigen-specific antibodies confirmed that rEg.P29 effectively activated immune responses (inducing antigen-specific IgG) while maintaining normal body weight, body temperature, liver function, and kidney function, thereby validating its immunogenicity, safety, and tolerability.

Body weight and temperature (Yirga et al., 2024) are key physiological indicators in animal studies, reflecting growth, health, and overall physiological status, and are critical for experimental evaluation (Matics et al., 2021; De Prekel et al., 2024). Stable body weight and temperature throughout the experiment indicated that rEg.P29 immunization did not compromise normal growth, consistent with safety evaluations of other vaccines. Adverse physiological stress (e.g., external stimuli) typically increases metabolism and energy consumption and may result in weight loss or growth arrest. Previous studies on avian vaccines (Kaab et al., 2018) have shown that effective immunization does not induce significant physiological stress. Stosman et al. (2022) reported no abnormalities in body weight, temperature, or food intake in mice immunized with a recombinant tuberculosis vaccine, further supporting vaccine safety with respect to growth indicators. In this study, sheep immunized with rEg.P29 exhibited stable appetite, mental status, body weight, and temperature comparable to PBS controls, confirming no adverse effects on growth and supporting its clinical safety and tolerability.

Antigen-specific antibodies are critical indicators for evaluating vaccine efficacy and safety, directly reflecting the intensity of the humoral response post-immunization and guiding vaccine development and clinical translation. In this study, immunization with rEg.P29 combined with the Quil A adjuvant resulted in a significant increase in antigen-specific IgG levels at 20 weeks post-immunization, whereas no significant changes were observed in specific IgA and IgM levels. These findings indicate that Quil A is essential for inducing a robust IgG response and highlight the critical role of adjuvant selection in the development of subunit vaccines against echinococcosis. Notably, the elevated IgE levels observed represent a predictable adaptive immune response triggered by parasitic antigens and adjuvants. Parasitic antigens are prone to inducing IgE production, while the QuilA adjuvant further promotes B-cell class switching toward IgE. Collectively, these factors lead to increased IgE levels, which theoretically raises the potential risk of type I hypersensitivity following booster immunization or antigen re-exposure. Nevertheless, no allergic clinical symptoms were observed throughout the study, confirming the safety of the vaccine under the current dose and immunization regimen. The rise in IgE was mainly attributed to the QuilA adjuvant, and optimizing adjuvant formulations to reduce IgE secretion could further improve vaccine safety in future research. Collectively, these results demonstrate that the rEg.P29 + Quil A formulation achieved a favorable balance between immunogenicity and safety, supporting its further development as a promising vaccine candidate against echinococcosis. Consistent with previous findings (Wang et al., 2016; Yang et al., 2023), our group previously demonstrated that rEg.P29 combined with Freund’s complete/incomplete adjuvant induced elevated IgG (including IgG1 and IgG2) and IgE levels in sheep following immunization and *Echinococcus granulosus* infection.

Routine blood and biochemical analyses complement each other in vaccine safety evaluation (Aldali et al., 2023) by monitoring systemic effects at different levels. Routine blood parameters (white blood cell count and differentiation, red blood cells, and platelets) reflect immune response intensity, inflammation, and infection risk (Olivieri et al., 2022; Worku et al., 2022). In this study, no intergroup differences were observed, confirming that rEg.P29 does not induce abnormal hematopoietic cell proliferation or consumption and supports a specific immune response without non-specific inflammatory activation. Stable lymphocyte counts suggest B-cell-mediated antibody production rather than non-specific proliferation. Normal red blood cell and platelet parameters exclude hemolysis or coagulation disorders, in contrast to hematological disturbances observed during natural *Echinococcus granulosus* infection (Wang et al., 2016), highlighting the safety advantage of recombinant antigens. Similar findings have been reported in studies on probiotic-adjuvanted anticoccidial vaccines in broilers (Arczewska-Włosek et al., 2022) and in preclinical evaluations of PCSK9 vaccines in mice (Momtazi-Borojeni et al., 2023), where no abnormal hematological or biochemical changes were observed.

Biochemical analyses focused on liver function, kidney function, and protein metabolism (Aldali et al., 2023). In the present study, no intergroup differences were observed. Notably, despite significant increases in specific antibody levels, total protein and globulin levels showed no statistical differences, which may be attributed to two factors: (1) globulins (Piza et al., 2021; Gori et al., 2022) represent a heterogeneous protein family (only 30%–40% of which are antibodies), and increases in specific antibodies may fall within normal physiological variation; and (2) significant globulin elevation typically occurs in chronic severe infections or autoimmune diseases (Grama et al., 2023; Lin et al., 2025). In this study, the observed immune response represents a physiological response following immunization and did not reach the threshold required to alter total protein and globulin levels. As a central organ involved in metabolism and immune regulation, the stability of liver-related indicators suggests that rEg.P29 immunization does not induce hepatocellular damage or impair synthetic function.

Studies evaluating different antigenic vaccine combinations for bovine-derived Neospora caninum have shown that multi-antigen formulations can induce stronger immune responses and improved protection, providing a potential direction for vaccine optimization (Tang et al., 2025). In contrast, studies assessing COVID-19 vaccines in normal and diabetic rat models have demonstrated vaccine safety under different physiological conditions through the analysis of biochemical and histopathological parameters, with no evidence of liver or kidney damage (Teymoorzadeh et al., 2023).

Our findings demonstrate that rEg.P29, as a parasitic antigen, induces specific immune responses without disrupting normal physiological metabolism. The stability of kidney function indicators further indicates that rEg.P29 immunization did not cause abnormal renal excretion, functional impairment, or altered protein metabolism due to immune stress. Similar results have been reported in studies of anticoccidial vaccines in chickens infected with Eimeria (Arczewska-Włosek et al., 2023), where recombinant antigens maintained a balance between immune activation and physiological stability.

Histopathological examination provides cellular- and tissue-level morphological data for assessing vaccine safety (Umer et al., 2020), offering direct insight into the effects of vaccination on key organs, such as the liver, spleen, and kidney, and enabling the identification of pathological changes, including inflammatory infiltration, cell necrosis, and fibrosis. In this study, H&E staining at 20 weeks post-immunization revealed intact tissue architecture in all groups, with no obvious abnormal histopathological changes or significant pathological damage observed within the observation period. As the central organ involved in immune responses, the spleen serves as a key indicator of vaccine-induced immune activation. Compared with the PBS control group, the rEg.P29 + QuilA co-immunization group exhibited extensive white pulp hyperplasia, densely packed lymphoid follicles, and enlargement of the periarteriolar lymphoid sheaths. These changes indicate effective vaccine-induced lymphocyte proliferation and activation, suggesting the induction of a humoral immune response.

This study confirmed that rEg.P29 immunization induces targeted immune responses with stable hematological and biochemical parameters, validating its safety and efficacy. Despite these findings, several limitations should be acknowledged. The study did not assess lymphocyte transformation efficiency, which reflects lymphocyte proliferation and functional activity, nor were oxidative stress markers (Peng et al., 2023) evaluated to detect subtle tissue damage. This study focused on the immune response and hematological and biochemical safety profiles induced by rEg.P29 immunization, without performing parasite challenge trials and protective functional verification. The protective efficacy of rEg.P29 in sheep has been well documented in previous studies; thus, we did not repeat the challenge experiment in the present work. Nevertheless, antibody elevation alone is insufficient to fully determine vaccine protective potential. Further challenge infection and functional assays are still required to complement the efficacy evaluation of this vaccine candidate. Additionally, the monitoring duration was relatively short, the sample size was limited, and the sheep population lacked diversity in age, body weight, and sex. Another limitation is the lack of a positive control group, as only Quil A adjuvant control was used. This design was intended to exclude adjuvant interference, given that the study’s primary focus was safety evaluation rather than efficacy assessment, and this limitation is acknowledged here to guide future studies. Future studies should expand the evaluation framework to include immune function parameters, hematological indices, and safety endpoints in a multidimensional approach. Long-term follow-up studies with larger and more diverse animal cohorts will improve statistical power and enhance the generalizability of the findings. These improvements will facilitate the clinical translation of the vaccine and provide more robust evidence to support its practical application.

In conclusion, this study demonstrates that rEg.P29 immunization induces robust and sustained antigen-specific IgG responses in sheep. Notably, histopathological examination revealed no vaccine-related damage to the liver, spleen, or kidney, which is consistent with the stability of hematological parameters (including immune cells, coagulation, and oxygen transport) and biochemical indices (such as liver function, kidney function, and protein metabolism), collectively supporting the favorable safety profile of the vaccine. These findings confirm the safety of the recombinant antigen, with no evidence of hematotoxicity or metabolic disruption, and provide a valuable reference framework for the safety evaluation of parasitic recombinant antigen vaccines (Larson et al., 2021; Yih et al., 2021).

## Data Availability

The raw data supporting the conclusions of this article will be made available by the authors, without undue reservation.
